# Accuracy of volume measurements by a clinical spirometer in multiple veterinary hospitals

**DOI:** 10.3389/fvets.2024.1475401

**Published:** 2024-12-04

**Authors:** Mathieu Raillard, Martina Mosing, Anthea Raisis, Adam Auckburally, Karla Borland, Susana Canfrán, Frances Downing, Alejandra García de Carellán Mateo, Paul MacFarlane, William McFadzean, Tristan Merlin, Karine Portier, Josephine Robertson, Joao Henrique Neves Soares, Barbara Steblaj, Aurora Zoff, Olivier L. Levionnois

**Affiliations:** ^1^School of Veterinary Medicine, College of Environmental and Life Sciences, Murdoch University, Murdoch, WA, Australia; ^2^Clinical Department for Small Animals and Horses, Veterinary University Vienna, Vienna, Austria; ^3^Southern Counties Veterinary Specialists, Ringwood, United Kingdom; ^4^Anderson Moores, Hursley, United Kingdom; ^5^Hospital Clínico Veterinario Complutense, Universidad Complutense de Madrid, Madrid, Spain; ^6^Davies Veterinary Specialists, Herts, United Kingdom; ^7^Hospital Veterinario de la Universidad Católica de Valencia “San Vicente Mártir”, Valencia, Spain; ^8^Langford Vets, University of Bristol, Langford, United Kingdom; ^9^Cave Veterinary Specialists, Wellington, United Kingdom; ^10^Eastcott Veterinary Clinic and Hospital, Swindon, United Kingdom; ^11^VetAgro Sup (Campus Vétérinaire), Centre de Recherche et de Formation en Algologie Comparée (CREFAC), University of Lyon, Marcy l’Etoile, France; ^12^Université Claude Bernard Lyon, Centre de Recherche en Neurosciences de Lyon, INSERM, CRNL, Trajectoire, Lyon, Bron, France; ^13^Small Animal Hospital, University of Glasgow, Glasgow, United Kingdom; ^14^Department of Surgical and Radiological Sciences, School of Veterinary Medicine, University of California, Davis, Davis, CA, United States; ^15^Section Anaesthesiology, Department of Diagnostics and Clinical Sciences, Vetsuisse Faculty, University of Zürich, Zürich, Switzerland; ^16^North Downs Specialist Referrals, Bletchingley, United Kingdom; ^17^Division of Anaesthesiology and Pain Therapy, Vetsuisse Faculty, University of Bern, Bern, Switzerland

**Keywords:** spirometry, D-lite, Pedi-lite, tidal volume, compliance, respiratory mechanics, accuracy, veterinary anaesthesia

## Abstract

**Introduction:**

Spirometry devices, which are components of many anaesthesia machines, are commonly used to assess lung mechanics during anaesthesia. Spirometry calibration usually adheres to manufacturer recommendations without established guidelines. Although more accurate and less variable than inbuilt spirometry in certain General Electric anaesthesia ventilators, near-patient spirometry lacks adequate evaluation.

**Methods:**

We assessed near-patient spirometers’ performance using Pedi-lite and D-lite flow sensors. Certified 1 L calibration syringes were used on 67 monitors located in 14 veterinary hospitals. Three consecutive inspired and expired volume values displayed by the monitors for each volume of the calibration syringe were recorded. Volumes studied were 50, 100, 150, 250, 300 mL for Pedi-lite and 150, 300, 450, 500, 750 mL for D-lite. Measured and targeted volumes were averaged, agreement error calculated. Accuracy was assessed plotting agreement errors against calibration volumes. A linear mixed-effects model was used to obtain linear regression between the error and the calibration volume. Mean, differential and proportional bias, limits of agreement, claimed accuracy and 10% clinical tolerance were calculated and displayed. Differences among monitors were evaluated using the Friedman rank sum test, differences between inspired and expired volumes using the Wilcoxon signed-rank.

**Results:**

Inter-monitor variability for inspired and expired volume readings using both sensors was high; intra-monitor variability was low. The error magnitude was independent of volumes evaluated. Using Pedi-lite, only a minority of measurements met manufacturer’s specification or a 10% clinical tolerance; both inspired and expired volumes were significantly underestimated. Using D-lite, superior performance was demonstrated for volumes between 300 and 750 mL (mean biases close to zero and the majority of measurements meeting manufacturer’s specifications and clinical tolerance). The difference between measured inspired and expired volumes with both sensors was significant.

**Discussion:**

These results support caution when interpreting clinical measurements of lung volumes and mechanics in anaesthetised patients when using these sensors. This is particularly important in smaller patients where lung volumes are below 300 mL. Trends should be reliable.

## 1 Introduction

Guidelines from specialist societies in pulmonary physiology include strict and comprehensive quality assurance testing for spirometer use (1). The performance of portable spirometers commonly used for human pulmonary function testing in China has been documented. Only 3 of 10 spirometers tested met all standards of quality and performance evaluated using a flow/volume simulator (2). When spirometry is applied during general anaesthesia, calibration and functional tests are often limited to manufacturers’ recommendations. No formal guidelines have been defined by professional organisations. Limited information about the performance of spirometers used in human or small animal veterinary anaesthesia is available since the evaluation of the Dräger Spirolog I for clinical use by Chackrabarti and Loh (3). However, a more recent study reported that near-patient spirometry was more accurate and less variable than inbuilt spirometry using two different GE (General Electric) Aisys CS^2^ anaesthesia ventilators, particularly with smaller tidal volumes (4).

A variety of monitors are currently used in both human and veterinary anaesthesia that incorporate near-patient spirometry. Datex Ohmeda/GE Healthcare monitors were originally developed for human patients and are now among the most commonly available technologies in veterinary anaesthesia (5). Although various modules are available (e.g., “E-COV,” “E-CAiOV,” “E-CAiOVX,” “E-sCAiO”), working principles, specifications and algorithms are similar.

Datex Ohmeda / GE Healthcare respiratory modules use specific sensors called Pedi-lite and D-lite flow sensors, originally designed for paediatric and adult human patients, respectively. Both sensors measure kinetic (or dynamic) pressure during inspiration and expiration using a two-sided Pitot tube. Additionally, gas density is calculated in real-time based on the gas composition measured by the gas analyzer built into the monitor. Inspiratory and expiratory dynamic pressures, along with gas density, are applied to Bernoulli’s equation to obtain their respective flow velocities. Finally, inspiratory and expiratory flows are calculated by applying their respective flow velocities to the known cross-sectional area of each flow sensor. Inspiratory and expiratory volumes are then calculated by numerically integrating their respective flows over time (6).

Pedi-lite and D-lite flow sensors are reported by the manufacturer to work over a wide range of ambient temperature, pressure, and humidity (7). Pedi-lite manufacturer specifications state that it is capable of measuring flows between 0.25 to 25 L minute^−1^ and volumes between 5 and 300 mL in both directions, with a resolution of 1 mL, with a claimed accuracy of ±6% or ± 4 mL (whichever is the largest volume) after a 10 min warm-up with I:E (inspiratory: expiratory ratios) within 1:4.5 to 2:1 and respiratory rates between 4 and 70 movements per minute (7). D-lite manufacturer specifications state that it is capable of measuring flows between 1.5 to 100 L minute^−1^ in both directions and volumes between 150 and 2,000 mL, with a resolution of 1 mL, with a claimed accuracy of ±6% or ± 30 mL (whichever is the largest volume) after a 10 min warm-up with I:E (inspiratory/expiratory ratios) of 1:4.5 to 2:1 and respiratory rates between 4 and 35 movements per minute (7). In principle, these specifications make this technology suitable not only for most human patients, but also, in veterinary anaesthesia, for dogs and cats and certain other species commonly used in animal experimentation (e.g., rabbits, pigs, sheep, primates). However, the performance of this spirometry technology in monitors used clinically in veterinary health care facilities has not been reported.

The aim of this study was to evaluate the performance of the GE near-patient spirometry component of the respiratory module using Pedi-lite and D-lite flow sensors to quantify known volumes delivered by 1 L calibration syringes. Our hypothesis was that displayed tidal volumes would align with the performance claimed by the manufacturer (±6% or ±4–30 mL, whichever is larger).

## 2 Materials and methods

### 2.1 Study protocol

As this was a bench study, no specific ethical approval was necessary.

A total of 67 monitors belonging to 14 veterinary hospitals of six countries (Australia, France, Spain, Switzerland, the United Kingdom, the United States of America) were tested. A total of 36 monitors had been serviced 1–24 months before data collection, though not specifically prior to or for this study. The service and calibration status of the other monitors was unknown, but all appeared fully functional.

The data collection was completed by a designated investigator in each centre. Investigators received an email containing detailed instructions, a demonstration video and a standardised Excel file for data recording. Cells not to be filled were locked and password protected, so the document could not be altered. The investigators were asked to use a specific (in-house) identification for every monitor. The monitors were renamed from “AA” to “CO” in Excel for further analysis. Three certified 1 L calibration syringes were purchased (Hans Rudolf, 5540B Series 1 Liter Calibration Syringe with 200,266 Outlet Port, 22 mm outer diameter/15 mm internal diameter tapers). They were sent via courier in their original, protective, packaging from a centre to the next one. However, one centre owned a calibration syringe already and used that one. That syringe was checked for accuracy. The data collection was completed before the syringes required manufacturer re-calibration/re-certification (1 year).

The volumes evaluated were chosen by authors MR, OL and MM as considered clinically relevant. For the Pedi-lite, the volumes checked were 50, 100, 150, 250, and 300 mL. The authors considered that a variability >10% would make the monitor clinically irrelevant, therefore did not plan on investigating any volume below 40 mL. Furthermore, since only 1 L syringes were used, any volume below 50 mL was considered impractical (lack of adequate graduation) and wasn’t investigated. For the D-lite, the volumes checked were 150, 300, 450, 500, and 750 mL.

### 2.2 Detailed procedure

As per manufacturer recommendation, the monitors were turned on for a minimum of 10 min before the start of the data collection. The monitors were set for either the Pedi-Lite or the D-lite, depending on the flow-sensor used. The sensors used were re-usable in all cases. All but one centre used paediatric or adult flow sensors manufactured by GE Healthcare. One centre (Langford Vets) used identical paediatric or adult flow sensors but manufactured by Intersurgical (Intersurgical Ltf, Wokingham, UK). The cap of the calibration syringe (in place when the devices were not in use) was removed. The syringes were connected to an appropriate male–female (15/22 mm) connector. The desired volumes were set on the syringe, from the lowest to the highest. Before connecting the syringe to the sensor, instructions included the verification of the sensor used (e.g., integrity of the sensors and ports, free from moisture). The Pedi-Lite or D-Lite connectors were disconnected from the breathing system, if applicable, and connected to the calibration syringe. The goal was for the calibration syringe to mimic the animals’ (or patients’) position, so the connectors were appropriately oriented to ensure the gas direction was correct. Traction on the plunger would mimic the animal’s inspiration, successive pressure on the plunger would mimic expiration. For each volume assessed, prior to the measurements, the investigators were asked to pump in and out five times to get a good rhythm and regular flows. This was done to ensure that the movements were smooth and regular, and to check that every action on the syringe was associated with a reading on the monitors’ screens. Over-enthusiastic (excessively quick) pumping could have resulted in flows higher than the upper end of the range, particularly with Pedi-lite. Once stabilised, three consecutive values displayed by monitor as inspired and expired volumes for each volume of the calibration syringe was recorded (“Inspired 1,” “Expired 1”; “Inspired 2,” “Expired 2”; “Inspired 3,” “Expired 3”).

### 2.3 Data analysis

Statistical analysis was performed using R version 4.3.2 (8).

For each measurement, the average between measured and targeted volumes and the agreement error {[(measured volume − volume of the calibration syringe)/volume of the calibration syringe] × 100} were calculated in Excel. The package “readxl” was used to import the data in R (9).

For both “Inspired” and “Expired” volumes, agreement errors were plotted against calibration volumes to assess the accuracy of the devices using the Pedi-lite or D-lite flow sensors using the package “ggplot2” (10). A linear mixed-effects model using the “lmer” function from the “lme4” package was used to obtain linear regression between the error (dependent variable) and the calibration volume (predictor variable) (11), including random intercepts for both monitor and repetition to account for the crossed structure of the data. In order to compare measured volumes against reference, mean bias (mean agreement error) as well as differential and proportional bias (linear regression) were calculated and displayed. Limits of agreement (±1.96 σ) were calculated according to Bland and Altman (12) including calculation of the within-device standard deviation (s_w_). Limits of accuracy claimed by the manufacturer (±6% or 4 mL, whichever the largest for Pedi-lite and ±6% or 30 mL, whichever the largest for D-lite) and clinical tolerance, arbitrarily set by the authors at 10%, were also displayed on the graphs for comparison.

A boxplot of the intra-monitor variability was constructed (individual monitors on the x-axis, agreement error on the y-axis) using the “ggplot2” package. The Friedman rank sum test (included in base R, in the “stats” package) was used to evaluate whether there were significant differences among monitors (Pedi-lite or D-lite), for “Inspired” and “Expired” volumes separately.

The Wilcoxon signed-rank test (included in base R) was employed to examine the potential disparity between inspired and expired volumes for both Pedi-lite and D-lite.

Statistical significance was considered when *p* < 0.05.

## 3 Results

One of the centres did not use Pedi-lite flow sensors. Therefore, the tests were carried on 66 monitors using Pedi-Lite and 67 for D-lite.

In eight monitors, one investigator used 50, 150, 250, 500, and 750 mL for the D-lite flow sensor, instead of the 150, 300, 450, 500, and 750 mL tested. The 50 and 250 mL volumes were not analysed.

In one case, no measurement was displayed for the expired volumes in the D-lite for one of the 150 mL volume. In one case, no value was reported by the investigator for the inspired volumes in the D-lite for one of the 150 mL volume.

### 3.1 Pedi-lite, inspired volume

**Figure 1:**
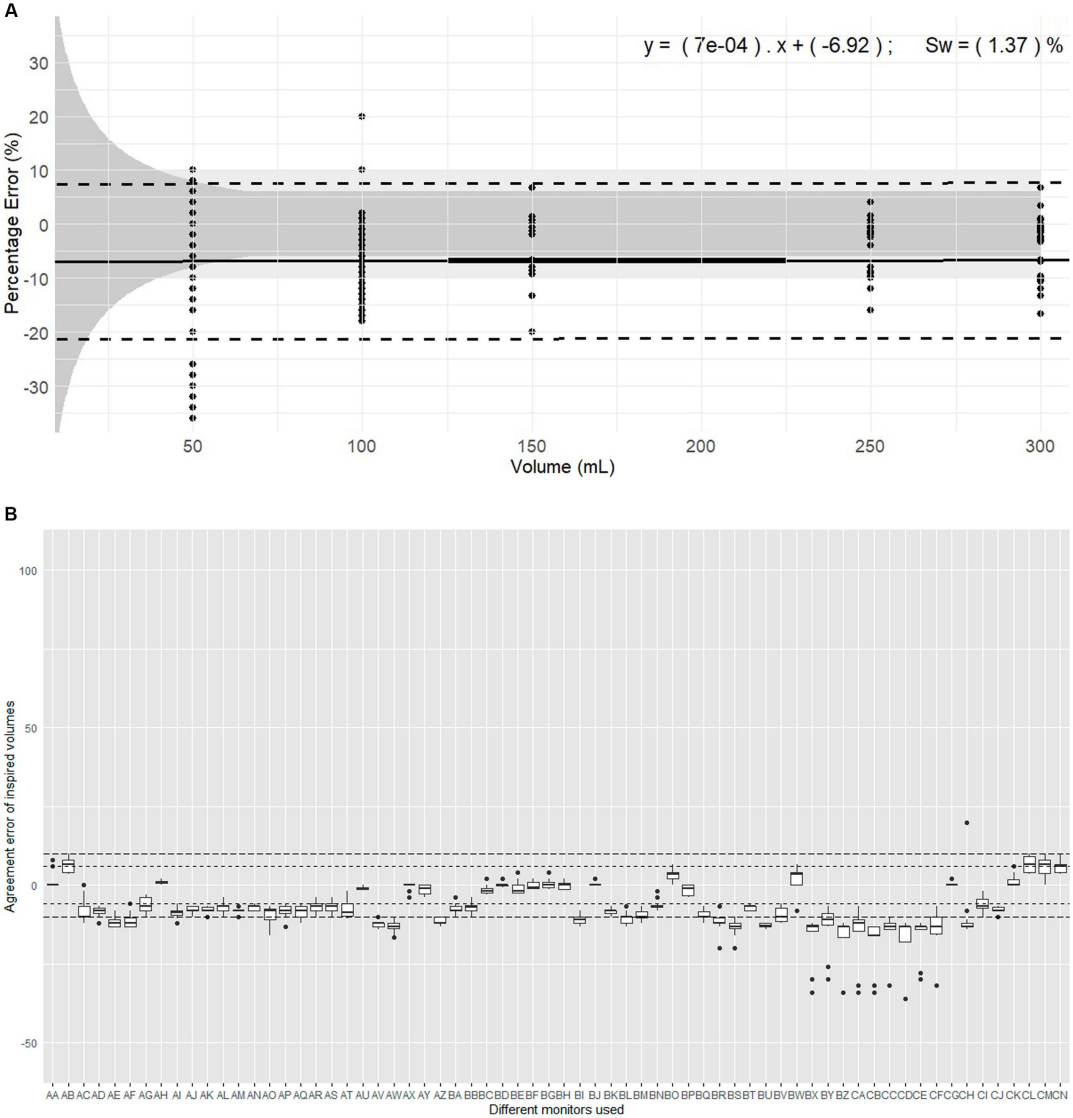
Evaluation of expired volumes pumped with a calibration syringe and displayed by 67 monitors belonging to 14 centres. These monitors are routinely used in clinical veterinary anaesthesia. **(A)** Illustrates the plot of Percentage errors (agreement error) against tested volumes for inspired volume with Pedi-lite. The solid line represents the overall bias, with the linear regression formula displaying differential and proportional components. The thicker solid segment in the middle of the graph shows the mean overall bias. The dotted lines represent the limits of agreement (±1.96 σ). The light grey area represents the clinical tolerance set at ±10%. The darker grey area represents the manufacturer’s claimed accuracy (“±6% or 4 mL, whichever the largest”). “s_w_” is the within-device standard deviation. **(B)** Represents the intra-monitor measurement variability for inspired volume measurements with Pedi-lite; the individual monitors are named from AA to CN; the dotted lines represent the 6% manufacturer’s specifications and 10% clinical tolerance.

The agreement error plot of the inspired volume for Pedi-lite, including the linear regression, is presented in Figure 1A. The mean bias was −6.81% showing a relevant underestimation, below the 6% claimed by manufacturers. The limits of agreement were ± 14.4%. Only 37% of single measurements were within manufacturer’s specifications (±6% or 4 mL); 59% were below, and 4% above. Additionally, 28% measurements were outside clinical tolerance limits (± 10% of the true volume) while 72% were acceptable.

Considering devices (Figure 1B), the mean bias of 47/66 monitors (71%) did not meet the manufacturer’s specifications and 20/66 (30%) were outside clinical tolerance. The Friedman rank sum test (Repeated measures) indicated a significant difference between monitors (Friedman chi-squared = 865.65, df = 65, *p* < 2.2*10^−16^). Within-device standard deviation (s_w_) was 1.37% showing good measurement consistency.

The linear mixed-effects model indicated no significant association between agreement error and the volume set on the calibration syringe. The proportional bias was 0.0007 showing minimal bias divergence over the range of measured volumes. Variability in the data was markedly influenced by the monitor, much less by repetition, with following substantial random effects variances: Monitor (40.9112 mL^2^, SD 6.3962 mL), Repetition (0.4778 mL^2^, SD 0.6913 mL), and Residual (13.5177 mL^2^, SD 3.6766 mL).

### 3.2 Pedi-lite, expired volume

**Figure 2:**
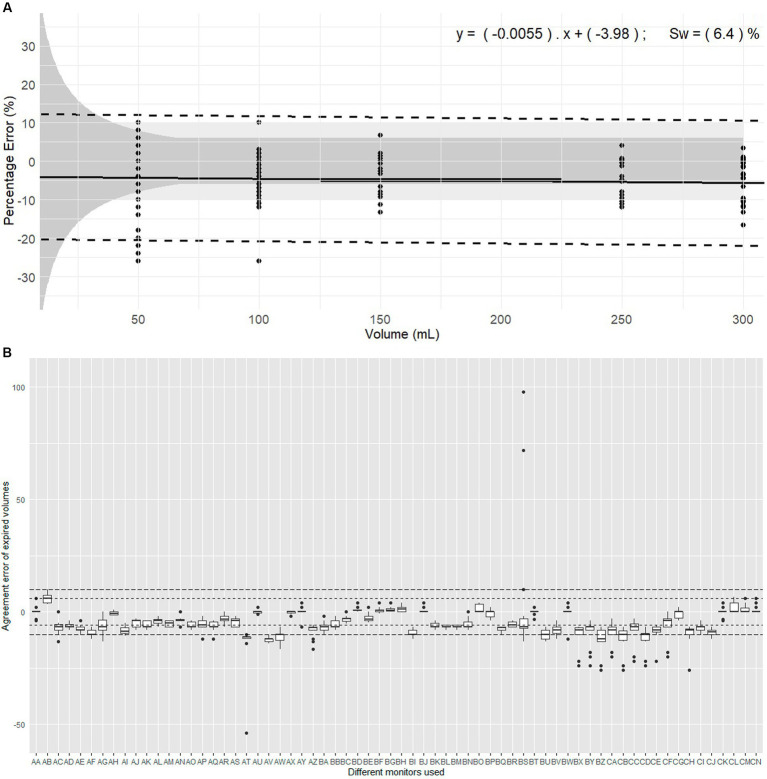
Evaluation of expired volumes pumped with a calibration syringe and displayed by 66 monitors belonging to 14 centres. These monitors are routinely used in clinical veterinary anaesthesia. **(A)** Illustrates the plot of Percentage errors (agreement error) against tested volumes for expired volume with Pedi-lite. The solid line represents the overall bias, with the linear regression formula displaying differential and proportional components. The thicker solid segment in the middle of the graph shows the mean overall bias. The dotted lines represent the limits of agreement (±1.96 σ). The light grey area represents the clinical tolerance set at ±10%. The darker grey area represents the manufacturer’s claimed accuracy (“±6% or 4 mL, whichever the largest”). “s_w_” is the within-device standard deviation. **(B)** represents the intra-monitor measurement variability for expired volume measurements with Pedi-lite; the individual monitors are named from AA to CN; the dotted lines represent the 6% manufacturer’s specifications and 10% clinical tolerance.

The agreement error plot of the expired volume for Pedi-lite, including the linear regression, is presented in Figure 2A. The mean bias was −4.91% showing a relevant underestimation. The limits of agreement were ±16.3%. Only 53% of single measurements were within manufacturer’s specifications (±6% or 4 mL); 46% were below, and 1% above. Additionally, 10% measurements were below clinical tolerance (±10% of the true volume) while 89% were acceptable.

Considering devices (Figure 2B), the mean bias of 33/66 monitors (50%) did not meet the manufacturer’s specifications and 8/66 (12%) were outside clinical tolerance. The Friedman rank sum test (Repeated measures) indicated a significant difference between monitors (Friedman chi-squared = 809.34, df = 65, *p* < 2.2*10^−16^). Within-device standard deviation (s_w_) was 6.40%.

The linear mixed-effects model indicated no significant association between agreement error and the volume set on the calibration syringe. The proportional bias was −0.0055 showing minimal bias divergence over the range of measured volumes. Intra-monitor variability over repetition was too small to be included in the model. The data was markedly influenced by the monitor with substantial random effects variances: Monitor (20.870 mL^2^, SD 4.568 mL), and Residual (26.78 mL^2^, SD 5.174 mL).

### 3.3 Comparison inspired and expired volumes with Pedi-lite

Overall with Pedi-lite, the inspired volume was 1.14% [0.00–4.26] smaller than the expired volume. The Wilcoxon signed-rank test revealed a statistically significant difference (V = 50,724, *p* < 2.2*10^−16^).

### 3.4 D-lite, inspired volume

**Figure 3:**
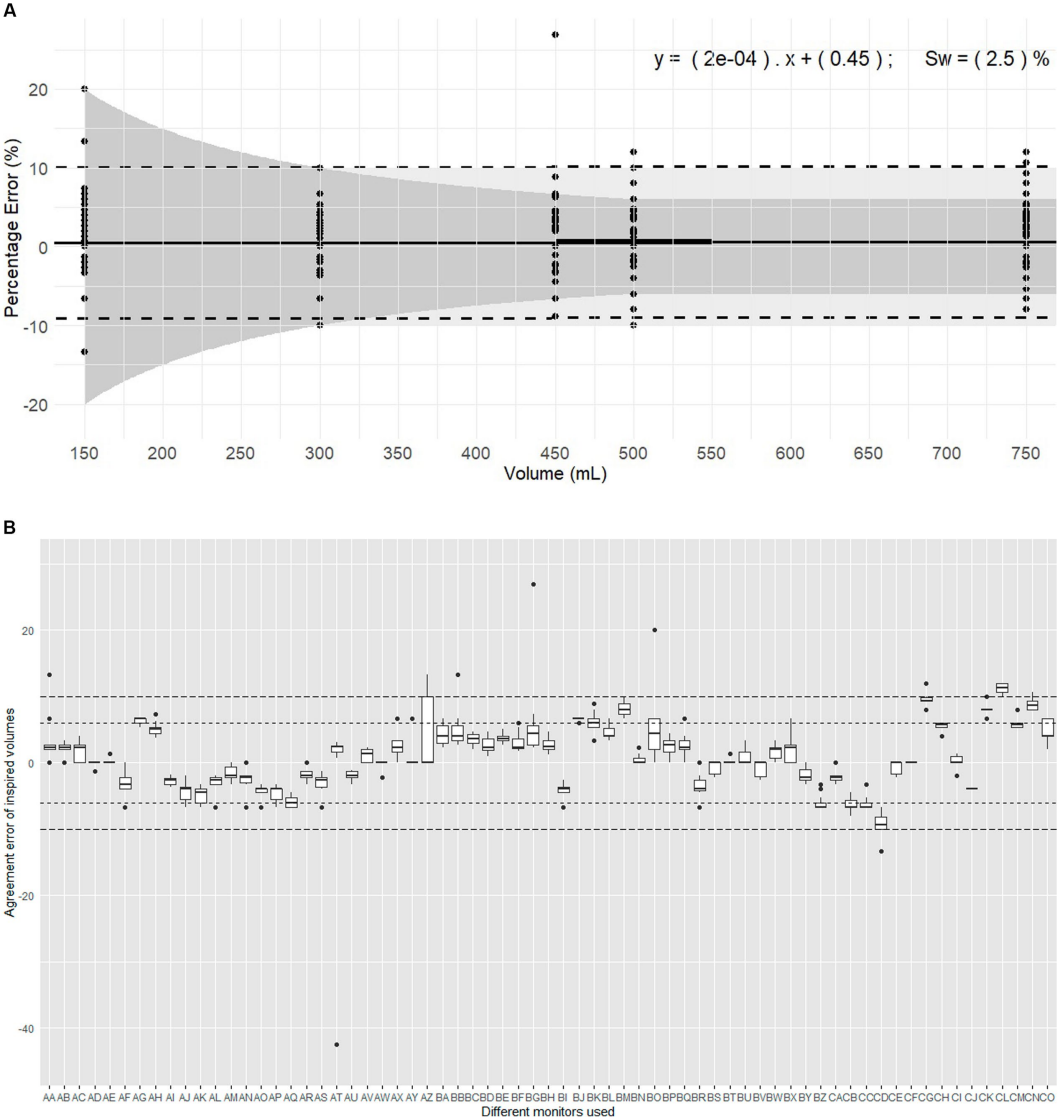
Evaluation of inspired volumes pumped with a calibration syringe and displayed by 67 monitors belonging to 14 centres. These monitors are routinely used in clinical veterinary anaesthesia. **(A)** Illustrates the plot of Percentage errors (agreement error) against tested volumes for inspired volume with D-lite. The solid line represents the overall bias, with the linear regression formula displaying differential and proportional components. The thicker solid segment in the middle of the graph shows the mean overall bias. The dotted lines represent the limits of agreement (±1.96 σ). The light grey area represents the clinical tolerance set at ±10%. The darker grey area represents the manufacturer’s claimed accuracy (“±6% or 4 mL, whichever the largest”). “s_w_” is the within-device standard deviation. **(B)** Represents the intra-monitor measurement variability for inspired volume measurements with D-lite; the individual monitors are named from AA to CO; the dotted lines represent the 6% manufacturer’s specifications and 10% clinical tolerance.

The agreement error plot of the inspired volume for D-lite, including the linear regression, is presented in Figure 3A. The mean bias was 0.56%. The limits of agreement were ± 9.6%. The majority of single measurements (94%) were within manufacturer’s specifications (±6% or 30 mL); 2% were below, and 4% above. Moreover, 98% measurements were within clinical tolerance (±10% of the true volume).

Considering devices (Figure 3B), the mean bias of 55/67 monitors (82%) and 66/67 monitors (99%) met the manufacturer’s specifications and the clinical tolerance, respectively. The Friedman rank sum test (Repeated measures) indicated a significant difference between monitors (Friedman chi-squared = 838.84, df = 66, *p* < 2.2*10^−16^). Within-device standard deviation (s_w_) was 2.50% showing good measurement consistency.

The linear mixed-effects model indicated no significant association between agreement error and the volume set on the calibration syringe. The proportional bias was −0.0009308 showing minimal bias divergence over the range of measured volumes. While the volume set did not significantly predict the error, the model identified substantial variability influenced by the monitor and repetition factors. Notably, monitor exhibited a variance of 18.8787 mL^2^ (SD 4.345 mL), repetition had a variance of 0.0351 mL^2^ (SD 0.1874 mL), and residual variance was 6.2178 mL^2^ (SD 2.4936 mL).

### 3.5 D-lite, expired volume

**Figure 4:**
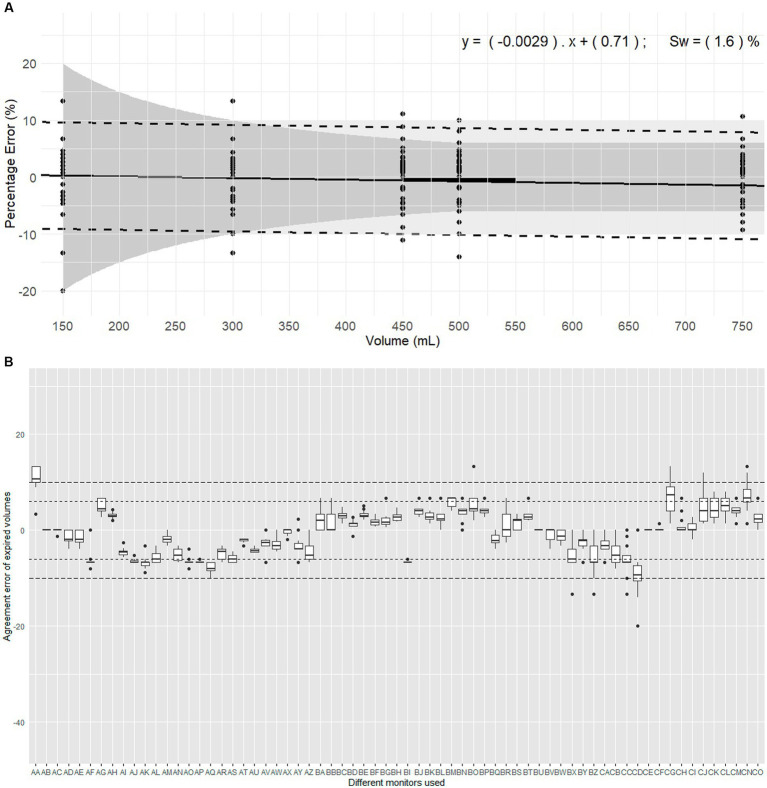
Evaluation of expired volumes pumped with a calibration syringe and displayed by 67 monitors belonging to 14 centres. These monitors are routinely used in clinical veterinary anaesthesia. **(A)** Illustrates the plot of Percentage errors (agreement error) against tested volumes for inspired volume with Pedi-lite. The solid line represents the overall bias, with the linear regression formula displaying differential and proportional components. The thicker solid segment in the middle of the graph shows the mean overall bias. The dotted lines represent the limits of agreement (±1.96 σ). The light grey area represents the clinical tolerance set at ±10%. The darker grey area represents the manufacturer’s claimed accuracy (“±6% or 4 mL, whichever the largest”). “s_w_” is the within-device standard deviation. **(B)** Represents the intra-monitor measurement variability for expired volume measurements with D-lite; the individual monitors are named from AA to CO; the dotted lines represent the 6% manufacturer’s specifications and 10% clinical tolerance.

The agreement error plot of the expired volume for D-lite, including the linear regression, is presented in Figure 4A. The mean bias was −0.52%. The limits of agreement were ±9.4%. The majority of single measurements (94%) were within manufacturer’s specifications (±6% or 30 mL); 5% were below, and 1% above. Moreover, 97% measurements were within clinical tolerance (±10% of the true volume).

Considering devices (Figure 4B), the mean bias of 54/67 monitors (81%) and 66/67 monitors (99%) met the manufacturer’s specifications and the clinical tolerance, respectively. The Friedman rank sum test (Repeated measures) indicated a significant difference between monitors (Friedman chi-squared = 827.31, df = 66, *p* < 2.2*10^−16^). Within-device standard deviation (s_w_) was 1.60% showing good measurement consistency.

The linear mixed-effects model indicated no significant association between agreement error and the volume set on the calibration syringe. The proportional bias was −0.0033843 showing minimal bias divergence over the range of measured volumes. Variability in the data was significantly influenced by the monitor and the repetition, with substantial random effects variances: Monitor (18.71251 mL^2^, SD 4.326 mL), Repetition (0.06605 mL^2^, SD 0.257 mL), and Residual (2.10106 mL^2^, SD 1.450 mL).

### 3.6 Comparison inspired and expired volumes with D-lite

Overall with D-lite, the expired volume was 1.39% [0.00–3.30] smaller than the inspired volume. The Wilcoxon signed-rank test revealed a statistically significant difference (V = 196,113, *p* < 2.2*10^−16^).

## 4 Discussion

In this study, we evaluated the performance of near-patient GE spirometry monitors (respiratory modules) originally designed for human anaesthesia and commonly used in veterinary practice. Overall, for all inspired and expired volumes measured using both Pedi-lite and D-lite sensor, there was a high variability in the readings, mostly between monitors, while intra-monitor variability was low. The magnitude of the error was independent of volumes set on the calibration syringe. Using the Pedi-lite flow sensor, only a minority of measurements fell within the manufacturer’s specification or a 10% clinical tolerance set by the authors. Both inspired and expired volumes were significantly underestimated. In contrast, when the D-lite flow sensor was used, a superior performance was demonstrated, with mean biases close to zero and the majority of measurements meeting manufacturer’s specifications and clinical tolerance. Nonetheless, this heightened accuracy was only valid within the volume range of 300 to 750 mL as the expected accuracy is out of ±10% below 300 mL with D-lite and volumes above 750 mL were not investigated. Furthermore, there was a significant difference between measured inspired and expired volumes with both Pedi-lite and D-lite.

A previous study investigated the difference between inspired and expired tidal volumes measured with near-patient or inbuilt spirometry during ventilation of a paediatric lung simulator (4). The GE E-sCAiOVE module was used as “near-patient” monitor and the GE Aisys CS^2^ anaesthesia ventilator was used as the “inbuilt” spirometry monitor. Overall, the variability in measured volumes was smaller with near-patient than with inbuilt spirometry. Therefore, the authors recommended the use of near-patient spirometry over inbuilt spirometry. The same near-patient technology was used in our study. Morgenroth et al. reported some variability in measured volumes, which our results confirmed (4). However, unlike in our study, the variability reported decreased with increasing volumes. Our results highlighted a high variability throughout the range of volumes measured, without any association between increasing volume and reducing variability of the measurements. This could be explained by the marked inter-monitor variability and the fact that only two monitors were used in the previous study and 67 in ours. Our results bring additional insights about the performance of individual near-patient spirometry monitors. Given the significant variability observed between monitors, we recommend that each spirometry monitor used in clinical practice undergo individual assessment. If the bias and accuracy of monitors are not acceptable to the clinicians using them, correction factors could be applied if necessary. However, given the relatively low intra monitor variability, the assessment of trends should be relatively reliable.

A 2022 survey involving 128 veterinary anaesthetists and criticalists revealed that spirometry was employed in veterinary anaesthesia (5). More than three-quarters of respondents deemed spirometry essential in either “selected” (43%) or “most” cases (33%). Pressure-volume loops emerged as the most widely used display. The specific monitoring of compliance and resistance of the respiratory system was also frequently reported (5). Based on the present study’s results, the authors recommend a cautious interpretation of the flow-volume and pressure-volume loops, as well as variables derived from volumes measurements (i.e., compliance, resistance, that are calculated using expired tidal volume values (7)), in particular with the Pedi-lite flow sensor and for volumes below 300 mL with the D-lite flow sensor.

The difference between inspired and expired volumes has been used as a method to evaluate the presence/absence of leak around tracheal tubes (13). Given the results of the present study, a part of the difference between inspired and expired volumes may come from measurement error and may need to be considered when evaluating the presence of a leak with near-patient GE spirometry monitors. Previous control and calibration of the modules may help preventing incorrect interpretation.

Selected specifications from the manual of use and results of this study are combined in Table 1, offering a practical reference for clinicians using or having to choose between Pedi-lite or D-lite sensors. The authors determined a clinically appropriate tolerance of ±10% (arbitrarily). Notably, recent manuals of use provided by the manufacturer exclude tracheal tube diameter consideration, while earlier versions of the manual mentioned them. Patient respiratory modules evolved from two slots (wider) to one slot (smaller) modules. However, the working principles and algorithms used in the spirometry component of the modules were unchanged. Therefore, the tool presented in Table 1 is presented irrespective of tracheal tube diameters. In humans, more consistency in the diameter of ETT used is expected. However, due to the large variation in species and breeds in veterinary practice, this factor requires further investigation.

**Table 1:** Practical reference for clinicians using or having to choose between Pedi-lite or D-lite sensors.

Tidal volume (mL)	Pedi-lite, inspired volume^*^	Pedi-lite, expired volume^*^	D-lite, inspired volume^**^	D-lite, expired volume^**^
<5 mL				
5–40 mL	Accuracy ±10–80%	Accuracy ±10–80%		
40–150 mL	Underestimation; Monitors limits of agreement out of manufacturer specifications and of the 10% clinical tolerance	Underestimation; Monitors limits of agreement out of manufacturer specifications and of the 10% clinical tolerance		
150–300 mL	Underestimation; Monitors limits of agreement out of manufacturer specifications and of the 10% clinical tolerance	Underestimation; Monitors limits of agreement out of manufacturer specifications and of the 10% clinical tolerance	Accuracy ±10–20%	Accuracy ±10–20%
	Notes: Sensor may increase resistance			
300–500 mL			Accuracy ±6–10% (manufacturer) Low bias, limits of agreement within the 10% clinical tolerance	Accuracy ±6–10% (manufacturer) Low bias, limits of agreement within the 10% clinical tolerance
500–750 mL			Low bias, limits of agreement within the 10% clinical tolerance	Low bias, limits of agreement within the 10% clinical tolerance
750–2,000 mL			Not tested	Not tested
>2,000 mL				

This reference is a combination of selected specifications from the manual of use of the GE respiratory modules (7) and the results of the performance assessment of 67 spirometry monitors belonging to 14 veterinary institutions in which volumes displayed by monitors using Pedi-lite and D-lite flow sensors were compared to volumes of air administered with calibration syringes. The authors arbitrarily determined a clinically appropriate tolerance of ±10%.

* I:E 1:4.5 to 2:1; f_R_ 4 to 70 movements minute^−1^.

** I:E 1:4.5 to 2:1; f_R_ 4 to 35 movements minute^−1^.

This study has several limitations. Although the measurement method was standardised, the syringes were used by different investigators in each centre. Therefore, intraobserver and interobserver variability cannot be ruled out. In addition, we did not pre-test nor verify the syringes’ accuracy over time. Deterioration of Syringes over time, although unlikely, cannot be accounted for (14).

The manufacturer of the calibration syringe states an accuracy of 0.5% of full scale. That means that volume accuracy measurements made with this syringe are within ±5 mL. Therefore, up to 5–10% of the error measured in the 50 and 100 mL could be attributable to the use of large (1 L) calibration syringes. Ideally, multiple calibration syringes in the appropriate volume range would have been used for testing at the smaller test volumes.

While Datex Ohmeda/GE Healthcare asserts that regular calibration is not necessary, the manual of use states that the flow calibration should be performed if the difference between inspiratory and expiratory volumes is permanent (15). This suggests that the monitors used in this study were not calibrated. Although many monitors had been recently serviced prior to data collection, they were not specifically calibrated for this study. Additionally, the sensors used were not new, and their duration of use was unknown. Both decisions were intentional, as most veterinary users are either unaware of their monitors’ status or rely on periodic servicing (5), and they use the sensors regardless of their duration of use, making this representative of real clinical practice.

The centres were situated in various geographical regions, each characterized by different climates. Datex Ohmeda / GE Healthcare’s specifications are valid at the following operating conditions: ambient temperature between +10°C and + 40°C, ambient pressure between 660 mbar and 1,060 mbar, and ambient humidity between 10% RH (Relative Humidity) and 98% RH, non-condensing (7). None of the centres operated out of those conditions. However, environmental factors (e.g., humidity, temperature, gas density) were not recorded. Therefore, their influence could not be investigated. From the manufacturer’s manual of use, they should have a minimal impact. This cannot be confirmed by this study. Since most monitors did not seem to behave as claimed by the manufacturer, the real impact of environmental factors requires confirmation.

The Pedi-lite and D-lite flow sensors were connected to calibration syringes and not to any breathing system or tracheal tube during the data collection. This differs from common clinical practice in anaesthesia. Given the marked variety of anaesthetic machines, breathing systems and tubes found in veterinary practice, the authors decided to standardise this aspect of the data collection. In addition, the authors considered that the volumes measured by a near-patient spirometry monitor should still be accurate even in animals disconnected from the breathing system. However, it could be speculated that this may induce a Venturi effect and alter gas flow, that could account for some of the difference between inspiratory and expiratory volumes. In addition, the expired gas composition *in vivo* is different than air and this could have influenced our results.

Finally, the findings are exclusively applicable to the GE near-patient respiratory modules using Pedi-lite and D-lite flow sensors. More research is needed to evaluate the performance of different devices used in (veterinary) anaesthesia.

In this field study, the accuracy of near-patient spirometry included in respiratory modules of GE monitors using the Pedi-lite flow sensor were not consistent with manufacturer’s claim and only a minority of measurements fell within the 10% clinical tolerance set by the authors. A superior performance was demonstrated when the D-lite flow sensor was used. However, this was only valid within the volume range of 300 to 750 mL. Despite low intra-monitor variability permitting the use of the monitors for trends, the inter-monitor variability was high suggesting a more regular need for check and calibration. As this seems to be uncommonly performed, especially in veterinary settings (5), a cautious interpretation of the flow-volume and pressure-volume loops, as well as variables derived from volumes measurements is recommended.

## Data Availability

The datasets analysed for this study can be found in the BORIS (Bern Online Repository and Information System): https://boris-portal.unibe.ch/entities/product/243d659d-7e8a-4799-840d-5aceffbb46e0.
